# Digital intervention in improving the outcomes of mental health among LGBTQ+ youth: a systematic review

**DOI:** 10.3389/fpsyg.2023.1242928

**Published:** 2023-09-22

**Authors:** Yanni Liu, Ying Cheng Wu, Hongpeng Fu, Wu Yuan Guo, Xukang Wang

**Affiliations:** ^1^McCormick School of Engineering, Northwestern University, Evanston, IL, United States; ^2^Tandon School of Engineering, New York University, New York, NY, United States; ^3^School of Law, University of Washington, Seattle, WA, United States; ^4^Department of Curriculum and Instruction, The Education University of Hong Kong, Hong Kong, Hong Kong SAR, China; ^5^Sage IT Consulting Group, Shanghai, China

**Keywords:** digital intervention, LGBTQ+, youth, mental health, systematic review

## Abstract

LGBTQ+ youth experience mental health disparities and higher rates of mental disorders due to barriers to accessing care, including insufficient services and the anticipated stigma of revealing their identities. This systematic review incorporated 15 empirical studies on digital interventions’ impact on LGBTQ+ youth mental health, examining their potential to address these inequities. This study innovatively categorized existing digital interventions into four streams: Structured Formal (telehealth, online programs), Structured Informal (serious games), Unstructured Formal (mobile applications), and Unstructured Informal (social media). We found that S&F and U&F effectively reduced symptoms. U&F showed potential but required enhancement, while U&I fostered resilience but posed risks. Further integration of emerging technologies like virtual reality may strengthen these interventions. This review identifies the characteristics of effective digital health interventions and evaluates the overall potential of digital technologies in improving LGBTQ+ youth mental health, uniquely contributing insights on digital solutions advancing LGBTQ+ youth mental healthcare.

## 1. Introduction

Lesbian, gay, bisexual, transgender, queer, and other gender and sexual minorities, collectively abbreviated as LGBTQ+, have been found to exhibit poorer mental health outcomes compared to their heterosexual and cisgender peers, with higher rates of depression, anxiety, and other mental health disorders (Borgogna et al., 2019). Specifically, research shows that sexual and gender minorities report depressive symptoms at 1.5 times the general population rate, while transgender individuals demonstrate even higher rates, with studies indicating that 59% of transgender participants experience clinically significant depression (Sutter and Perrin, 2016; Ancheta et al., 2021). However, LGBTQ+ youth face greater challenges in adolescence (Wilson and Cariola, 2020) and are thought to be at higher risk of generalized anxiety disorder and suicidality due to direct and indirect discrimination and harassment from unsupportive family, peers, and society at large (Kaniuka et al., 2019). The 2020 national survey of LGBTQ+ youth in the United States reported that 40% of LGBTQ+ individuals ages 13 to 24 seriously considered suicide plans, and 68% of them reported depression and anxiety symptoms (Delmonaco et al., 2022). Furthermore, despite experiencing the severity of psychological conditions, sexual and gender minorities face barriers to accessing safe and adequate mental health treatments and services due to various sociocultural factors like insufficient health resources designated for them as well as discrimination against their minority sexual orientation and gender identities (Gilbey et al., 2020). These barriers to help-seeking give rise to health disparities in this population. Consequently, reducing these disparities by improving access is crucial for advancing their overall health.

In addressing these issues, digital interventions that bypass the need for in-person contact (Rauschenberg et al., 2021), delivered via computers, smartphones, or other advanced technological tools such as wearable devices, are increasingly recognized for improving healthcare access and mitigating health inequalities (Friis-Healy et al., 2021). Such digital health interventions, unconfined by traditional clinical settings, offer benefits including convenience, 24/7 accessibility, and the avoidance of travel (Gilbey et al., 2020). The anonymity provided by these platforms could also minimize stigma, encouraging help-seeking for mental health difficulties. The evidence base for digital mental health interventions for general populations is vast and prior systematic reviews have suggested that these types of interventions could significantly reduce symptoms and improve psychological well-being in general populations (Firth et al., 2017; Lattie et al., 2019), a finding further supported by several studies that deemed such digital tools as feasible and acceptable for mental health applications (Lim et al., 2019; Bevan Jones et al., 2020; Rauschenberg et al., 2021). However, limitations exist as these reviews overlooked interventions’ characteristics and effective components, which could facilitate subsequent research to understand mechanisms, and leverage this knowledge to amplify effectiveness and maximize efficacy. Moreover, none centered specifically on the understudied LGBTQ+ population. Although recent systematic reviews demonstrate the efficacy of digital technologies for LGBTQ+ adults, they concentrated primarily on physical conditions like HIV prevention and sexual behaviors rather than mental health interventions for LGBTQ+ youth in particular (Bailey et al., 2015; Wadham et al., 2019; Gilbey et al., 2020). Evidence summarizing the use of digital technologies for mental health improvement among LGBTQ+ youth specifically is still exiguous, and the overall effectiveness of these innovations is also unclear despite recent surges in digital tools for this demographic. Consequently, an overview of tailored digital interventions for LGBTQ+ youth may inform further research on enhancing access to targeted services and resources, thereby reducing pre-existing mental health disparities.

A comprehensive overview of this rapidly expanding research area will enable future studies to identify gaps in intervention development and assess the overall strength of evidence supporting their use across the diverse spectrum of young LGBTQ+ populations. Accordingly, the primary objective of this review was to answer the following research questions: (1) What are the characteristics of evidence-based digital health interventions for improving mental outcomes, especially focusing on depression, anxiety, and stress in LGBTQ+ young people? (2) Are existing digital tools acceptable, feasible, and effective at decreasing specific symptoms? (3) What is the potential direction for further research to improve the existing digital tools and leverage the latest technologies for these mental health issues among this population?

## 2. Materials and methods

### 2.1. Literature search

**Figure 1:**
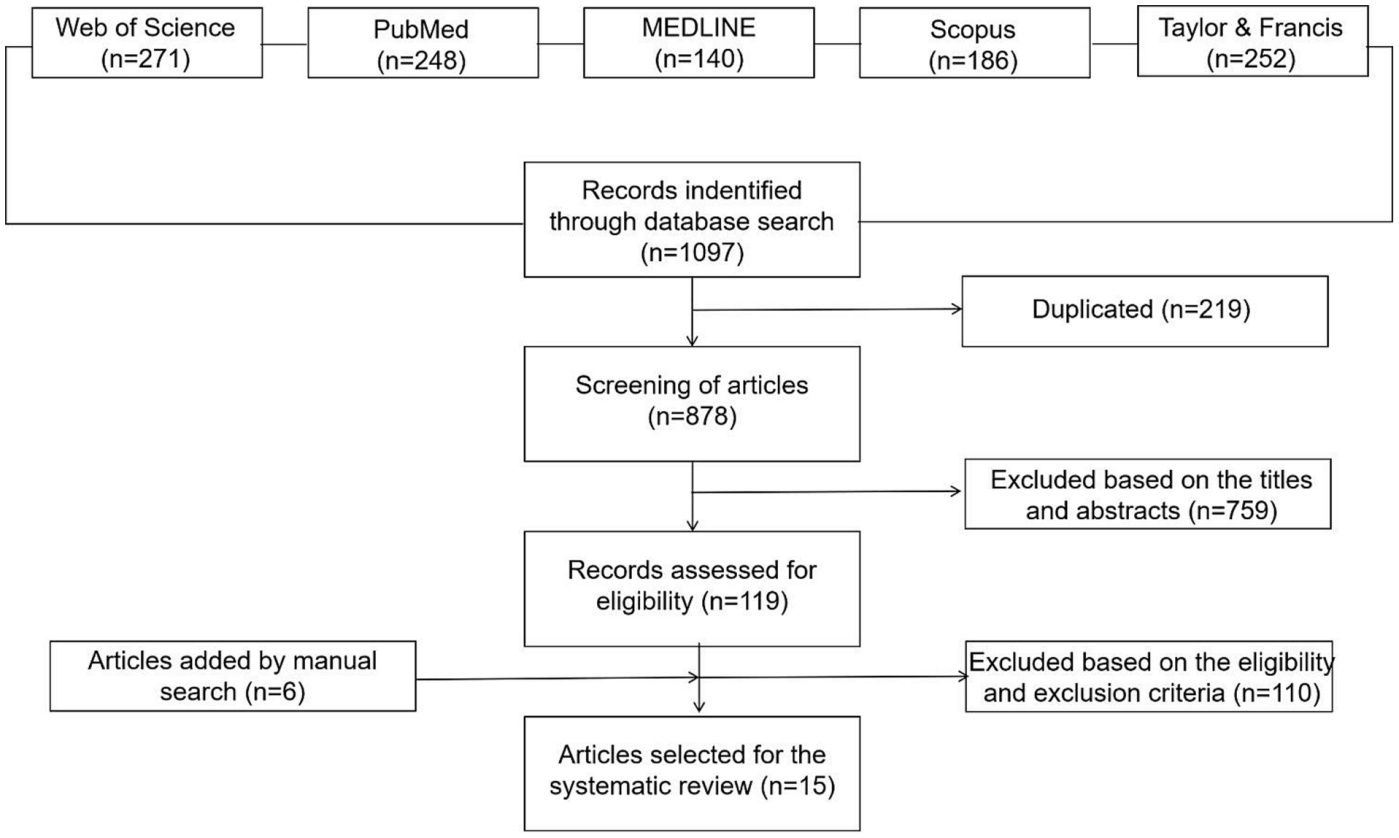
Flow diagram of the systematic review process.

This systematic review adhered to the Preferred Reporting Items for Systematic Reviews and Meta-Analyses (PRISMA) guidelines, conducting comprehensive searches across multiple databases including Web of Science (271), PubMed (248), Medline (140), Scopus (186), and Taylor & Francis (252) using specific keywords. The focus of this research was on young individuals within the LGBTQ+ community. To encompass this population, keywords were chosen that embodied the diversity of sexual orientations and gender identities, as well as those highlighting interventions involving digital technologies or platforms. Furthermore, keywords were utilized to identify study outcomes related to mental health, encompassing terms for psychological conditions and mental disorders linked with mental well-being. The full search strategy and list of keywords are detailed in Table 1. There were no temporal limitations applied during the database search, which was carried out on August 17, 2022. Beyond the primary database search, additional sources were incorporated into this review, selected based on citations within the articles of the primary research (Figure 1).

**Table 1:** The search strategy of systematic review.

Search query	Keywords (searched as titles, abstracts, and keywords in each database)
1	“LGBTQ” OR “LGBT” OR “lgbtq+” OR “sexual minority” OR “sexual minorities” OR “gender minority” OR “gender minorities” OR “lesbian” OR “gay” OR “bisexual” OR “transgender” OR “queer”
2	“youth” OR “young” OR “young adults” OR “young people” OR “adolescents” OR “students”
3	“mental” OR “mental health” OR “depression” OR “depressive” OR “anxiety” OR “well-being” OR “wellbeing” OR “disorder” OR “emotion” OR “suicide” OR “psychological”
4	“digital” OR “technology” OR “technologies” OR “internet” OR “internet-based” OR “web-based” OR “social media” OR “social network” OR “SNS” OR “telehealth” OR “ai” OR “vr” OR “eHealth” OR “telemedicine” OR “mHealth” OR “mobile phone”
5	“intervention” OR “treatment” OR “therapy” OR “implement” OR “application” OR “program” OR “strategy” OR “implementation”
Final search query	1 AND 2 AND 3 AND 4 AND 5

### 2.2. Inclusion and exclusion criteria

Article screening was conducted manually in a systematic three-stage process: (1) title screening; (2) abstract screening; and (3) full-text screening of the related studies. Articles were included in the review if: (a) they were empirical studies published as peer-reviewed journal articles; (b) their full text was in English language; (c) they reported at least one group of sexual or gender minorities as the targeted population of the intervention; (d) a substantial number of participants were aged 12–22, and at least 50% of the sample’s participants fell within the defined age group; (e) the interventions in studies was delivered entirely via digital mediums; (f) the studies focused exclusively on mental or psychological outcomes and described an evaluation of the implemented intervention.

Articles were excluded from the review under the following conditions: (a) if the article was a review, opinion, letter, or commentary rather than a peer-reviewed research article; (b) if the full text was not available in English; (c) if the research did not include any individuals identifying as sexual or gender minorities; (d) if the majority of the participants in the intervention group were older than 25, as the focus was on younger individuals; (e) if the intervention was non-digital, such as in-person therapy; (f) if the focus was on physical health outcomes (e.g., HIV), rather than mental health among the intervention recipients.

### 2.3. Construction of database and data analysis

In total, 1,097 published papers were potentially identified to be related to this systematic review before the screening process After removing 219 duplicates, we evaluated the titles and abstracts of 878 unique citations according to the inclusion and exclusion criteria of this review. Among those citations, 759 were excluded due to their irrelevancy to the criteria. Then, 119 research articles were retrieved for full-text screening. Because of exclusion criteria in 2.2, only 15 articles were included in the database of final review (Table 2).

**Table 2:** Summary of digital mental health interventions for LGBTQ+ Youth.

Categories of intervention	Intervention name	Intervention type	Digital platform	Study	Primary health outcome	Mean age (range)	Identification	Sample size	Study design	Study setting	Measured result
	Telehealth										
Structured and Formal (S&F)	AFFIRM Online	Affirmative cognitive behavioral therapy	Computer via Zoom	Craig et al. (2021b)	Depression	21.17 (14–29)	LGBTQA+ youth	78	Quantitative, RCT, acceptability test	US	Depressive symptoms↓ Stress appraisal ↑ 95.2% acceptability rate
	imi	Identity-affirming treatment	Web	Bauermeister et al. (2022)	Stress appraisals	16.5 (13–19)	Sexual and gender minority youth	270	Quantitative, Pilot RCT	US	Stress appraisal ↑ Depressive and anxiety symptoms
	Online program										
	QueerViBE	Interactive video tutorials	Web	Martin (2019)	Psychological well-being	18 (15–21)	Trans youth	156	Quantitative, RCT	UK	Psychological distress ↓ Stress appraisal ↑
	OMBSR/online mindfulness-based stress reduction	Self-regulation daily mindfulness activities	Web	Jabson Tree and Patterson (2019)	Perceived stress	18+	LGB	24	Quantitative, uncontrolled pilot	US	Perceived stress ↓
	Virtual Camp	Interactive video tutorials	Computer via Zoom	Gillig et al., 2022	Depression	15.5 (12–19)	LGBTQ youth	41	Quantitative, longitudinal survey	US	Depressive symptoms ↓
	Web & mobile app										
Unstructured and Formal (U&F)	Q Chat Space	Chat-based programs	Web	Fish et al. (2022)	N/A	(13–19)	LGBTQ youth	291	Qualitative, pilot study	US	High acceptability and feasibility rate
	TODAY!	Daily psychoeducation	Mobile application	Fleming et al. (2017)	Depression and anxiety	19 (18–20)	Young sexual minority men	9	Qualitative, usability testing	US	High acceptability rate
	Serious game										
Structured and Informal (S&I)	SPARX	Computerized cognitive behavioral therapy	Computer	Strauss et al. (2019)	Depression	15.6 (11–18)	Trans and gender diverse youth	14	Qualitative, focus group	AU	N/A	
				Lucassen et al. (2021)	Depression	(12–19)	Transgender and cisgender youth	9,079	Quantitative	NZ	Lack of change in depression for trans youth	
	Rainbow SPARX	Computerized cognitive behavioral therapy	Computer	Lucassen et al. (2015)	Depression	16.5 (13–19)	Sexual minority youth	21	Quantitative	NZ	Depressive symptoms ↓ Anxiety symptoms ↓	
				Lucassen et al. (2018)	Depression	17.9 (15–22)	LGBT+ young people	21	Qualitative, focus group	UK	Low rates of acceptability
	Social media and online groups										
Unstructured and Informal (U&I)	Social media	User experience	Web, mobile device	Craig et al. (2015)	Stress	19.47 (18–22)	LGBTQ young adults	19	Qualitative, grounded study	CA	Online media may be a catalyst for resilience among LGBTQ youth
		Experience of acceptance and hostility	Web, mobile device	Pellicane et al. (2021)	Depression and anxiety	19.87	LGB+ and heterosexual young adults	382	Quantitative, longitudinal study	US	Experiencing higher level of acceptance with lower depressive symptoms for LGB+ participants
	Facebook	Problematic social media experience	Web, mobile device	Vogel et al. (2021)	Depression	21.9 (18–25)	Sexual and gender minority youth	302	Qualitative, observational study	US	Problematic social media use would cause greater depressive symptoms
		Experience of victimization, cyberbullying, and online support	Web, mobile device	McConnell et al. (2017)	Psychological distress	24.02 (19–28)	LGBTQ young adults	175	Quantitative, longitudinal research	US	A sizable effect of cyberbullying on psychological distress but social integration was negatively associated with distress

We assigned a unique identification number to each selected publication in our database. Initially, one author recorded essential details for every paper: journal name, year of publication, paper title, digital object identifier (DOI), author names, research context, country of focus, focus group, research methods (e.g., qualitative, quantitative, and mixed methods), and sample size. Subsequently, this author conducted an in-depth analysis to glean additional crucial details, such as intervention types, intervention names, digital platforms, primary health outcomes, measured results, limitations, and future directions. Another researcher cross-verified this information for consistency and accuracy.

To provide a comprehensive understanding of the landscape of digital interventions for LGBTQ+ youth mental health, we conducted descriptive analyses. Specifically, these analyses focused on identifying the types of interventions, research contexts, research designs, data collection methods, sample sizes, analysis methods, focal countries, and key theories employed across studies. With this foundation, we proceeded to discuss the characteristics, acceptability, feasibility, and effectiveness of existing digital health interventions, also suggesting avenues for future improvements. Finally, we engaged in a detailed discussion on four key online service streams that target mental health outcomes.

## 3. Results

### 3.1. Characteristics of evidence-based digital health interventions

Current studies of evidence-based digital health interventions mainly include four types of digital interventions aimed at improving mental health outcomes among LGBTQ+ youth (refer to Table 2). The four types included structured formal methods like Telehealth, structured informal methods like serious games, unstructured formal interventions like online programs, and unstructured informal methods such as social media platforms. The various techniques provide different therapeutic modalities including cognitive-behavioral therapy, identity-affirming treatments, and mindfulness-based stress reduction activities.

Structured formal methods, such as Telehealth, are hallmarked by their high levels of control and standardization, rendering them ideal for delivering tailored, consistent, and controlled therapeutic interventions. These digital health interventions are akin to traditional face-to-face therapeutic modalities but are delivered digitally. Examples of such interventions include AFFIRM online and imi. These interventions have been successful in reducing depressive symptoms and improving stress appraisals among LGBTQ+ youth (Craig et al., 2021b; Bauermeister et al., 2022). They typically provide therapeutic modalities including cognitive-behavioral therapy, often augmented with supplementary digital resources.

Structured informal methods represent a novel approach to therapeutic interventions. This category includes serious games designed with the intent to provide therapy in a more engaging, interactive, and user-friendly manner. Serious games aim to embed therapeutic concepts within their gameplay, rather than an overtly didactic approach. For example, games may teach emotional regulation skills through immersive gameplay mechanics, and users proceed through game levels while practicing evidence-based coping strategies (Strauss et al., 2019). This informal setting provides a more relaxed and low-pressure environment for users to learn and apply therapeutic techniques.

Unstructured formal interventions like online programs offer a different mode of digital intervention. Although less standardized than their structured counterparts, these interventions offer greater flexibility and customization to the user, allowing for a more personalized therapeutic experience. For example, researchers tested two unstructured online programs – QueerViBE and OMBSR. QueerViBE applies discursive methods to help LGBTQ+ youth negotiate prejudice and reflect on experiences (Jabson Tree and Patterson, 2019; Martin, 2019). OMBSR is an 8-week mindfulness-based stress reduction program that includes meditation and applying mindfulness to daily living. Both of these have shown efficacy in reducing psychological distress and perceived stress. These programs often incorporate therapeutic techniques such as mindfulness-based stress reduction activities, discussion of experiences, and other evidence-based therapeutic strategies that users can explore at their own pace.

Lastly, unstructured informal methods, such as social media platforms, represent the least formal of the digital interventions. These platforms create space for LGBTQ+ youth to connect, share experiences, exchange information, and find support (Craig et al., 2021b; Pellicane et al., 2021). The peer-driven, community-oriented nature of interventions is unique, often providing identity-affirming treatments within a supportive community. Thus, unstructured informal interventions may catalyze resilience within LGBTQ youth. However, risks exist as demonstrated in the study where Facebook use exposed users to discrimination, hate speech, and cyberbullying, negatively impacting psychological health (McConnell et al., 2017).

### 3.2. Acceptability, feasibility, and effectiveness of current digital health interventions

The acceptability of digital interventions varies considerably. Telehealth interventions have found favor due to their structured approach and consistent therapeutic support (Craig et al., 2021b; Bauermeister et al., 2022). However, these require a reliable, high-speed internet connection, potentially limiting their accessibility and acceptability, especially in areas with limited internet infrastructure. On the other hand, online programs and social media platforms offer an environment that LGBTQ+ youth might find more familiar and accessible. For instance, a study has shown that social media platforms foster a sense of community among LGBTQ+ youth, thus enhancing their acceptability (Craig et al., 2021b).

Feasibility is another critical aspect of digital interventions. Structured formal methods such as Telehealth are feasible if the requisite technological infrastructure is available. However, in certain areas where high-speed internet connection is unavailable or unreliable, these may not be feasible (Craig et al., 2021b; Bauermeister et al., 2022). Unstructured formal methods such as online programs offer more flexibility and are feasible given their requirement for less stringent control, although they require active user engagement, which might be challenging to sustain over extended periods. Furthermore, while serious games like SPARX and Rainbow SPARX are appealing due to their interactive nature, the feasibility of their widespread implementation may be limited by factors such as costs associated with development, maintenance, and updates (Lucassen et al., 2015, 2021).

The effectiveness of digital interventions also varies. Studies have shown that Telehealth interventions such as AFFIRM Online and imi can effectively reduce depressive symptoms and improve stress appraisals (Craig et al., 2021b; Bauermeister et al., 2022). Unstructured formal methods like online programs such as QueerViBE and OMBSR have shown effectiveness in reducing psychological distress and perceived stress (Lucassen et al., 2015, 2021). However, maintaining long-term effectiveness can be challenging due to the need for consistent user engagement. Serious games have had mixed success (Lucassen et al., 2015, 2021). While they have shown potential in treating depressive and anxiety symptoms, the effectiveness varies across individuals. Lastly, social media platforms have both positive and negative impacts. While they can foster resilience and acceptance among LGBTQ+ youth, they also present risks such as cyberbullying and problematic use, potentially exacerbating depressive symptoms and psychological distress (Craig et al., 2021b).

### 3.3. Potential improvement for the design of the existing research

Among the 15 studies included in the review, only three were randomized controlled trials (RCTs), suggesting that the positive outcomes observed could be attributed to external factors rather than the proposed intervention (Martin, 2019; Craig et al., 2021b; Bauermeister et al., 2022). As such, further studies should consider mixed methods, including RCTs, pre-post evaluations, and interviews, to ascertain the acceptability, feasibility, and effectiveness of the intervention on mental health (Hariton and Locascio, 2018). The majority of studies primarily employed quantitative methods, providing concrete statistical data and objective measurements (Craig et al., 2021b). However, qualitative methods offered rich, detailed data on individual experiences (Fish, 2020). Researchers have highlighted the value of combining both quantitative and qualitative methodologies. Additionally, only three studies collected short-term follow-up data, typically spanning 3 months (Lucassen et al., 2015; Jabson Tree and Patterson, 2019; Martin, 2019). However, previous research suggests that promising short-term results may not equate to long-term success (Das et al., 2016), underlining the need for more studies to prioritize long-term follow-ups post-intervention.

The research design review revealed that most included studies had small sample sizes, though they reported positive outcomes, notably within the Structured and Formal Intervention, and Unstructured and Formal Intervention categories. These findings demonstrated significant mental health improvements and high acceptance and feasibility levels. However, few interventions specifically targeted LGBTQ+ youth of color or individuals facing racism, who encounter multiple stressors (Sutter and Perrin, 2016). All studies were conducted in developed countries, with only one rural program where health services and resources were lacking (Jabson Tree and Patterson, 2019). Thus, to increase the generalizability and diversity of future research, the implementation of larger sample sizes and broader geographical coverage is recommended. Also, the populations studied encompassed a diverse range of subgroups within the LGBTQ+ youth community, including LGBTQA+ individuals, sexual and gender minorities, transgender youth, LGB individuals, and young men who identify as sexual minorities. However, some groups, like non-binary youth, gender non-conforming youth, and LGBTQ+ youth of color, were underrepresented, indicating a crucial need for more inclusive research. Comprehensively representing the full diversity of LGBTQ+ youth identities is crucial to maximize the effectiveness of digital tools for diverse groups.

While digital interventions hold significant promise for improving mental health outcomes among LGBTQ+ youth, continued research is necessary to address potential risks and optimize these platforms for therapeutic use. Interventions such as AFFIRM online, imi, QueerViBE, and Virtual Camp have demonstrated effectiveness, while apps like Q Chat Space and TODAY! maintain high user acceptability. Games like SPARX offer mixed outcomes but show potential. Although social media and online platforms foster resilience and support, they come with risks like cyberbullying and increased distress. As digital tools evolve, it remains essential to balance their therapeutic potential with these challenges.

## 4. Discussion

Numerous studies have confirmed that LGBTQ+ youth experience exacerbated mental health challenges, including heightened levels of anxiety, depression, and other psychological disorders when compared to the broader population (Borgogna et al., 2019). The implementation of digital interventions could serve as a key component in addressing these disparities. The necessity for such internet-based assistance has become even more pronounced in the wake of COVID-19, a situation that has led to increased social isolation among these individuals who have not met with support from their families under stay-at-home guidelines (Salerno et al., 2020). This review aims to provide a comprehensive overview of the existing digital interventions for this demographic. It seeks to assess their overall acceptability, feasibility, and effectiveness, while also offering guidance and recommendations for future development.

Previous studies primarily depict “Structured Intervention (SI)” as a relatively novel method to instigate long-term external changes (Bloom and Cohen, 2007), intended to improve health behaviors and outcomes. This concept is often paralleled with professionally driven psychotherapy. On the contrary, “Unstructured Intervention (UI)” is generally defined as a self-governed rehabilitation approach, devoid of preset rules, focusing on individual needs and interests, with assistance from facilitators (Lai et al., 2022). To elaborate, SI relies on a treatment framework and goals devised by healthcare professionals, while UI, aimed at bolstering social support, can be executed through numerous strategies such as group activities and community assistance.

In addition, based on our interpretation, “Formal Intervention (FI)” pertains to collaborations with medical services and professional guidance. In contrast, “Informal Intervention (II)” involves processes where patients independently strive to enhance their health outcomes. Accordingly, in this discussion regarding online services for mental health outcomes, with a focus on depression, anxiety, stress, and distress, the prevalent research trend has bifurcated into four streams: Structured Formal Intervention, Structured Informal Intervention, Unstructured Formal Intervention, and Unstructured Informal Intervention.

### 4.1. Implication for structured and formal intervention (S&F)

S&F is recognized as one of the most effective digital interventions for enhancing mental health, primarily due to its combination of affirmative treatment and professional counseling (Lelutiu-Weinberger and Pachankis, 2017; Pachankis, 2018). The S&F intervention’s scope varies depending on the level of professional involvement, dividing it into two categories: telehealth (pi+), characterized by direct professional intervention, and online programs (pi-), consisting of self-directed or module-based interventions.

Based on the study’s suggestion that cognitive behavioral therapy (CBT) could be a gold standard treatment for multiple mental issues among LGBTQ+ young people (Hall et al., 2019), an affirmative CBT group intervention, AFFIRM, was constructed to address the dearth of evidence-based programs specifically designed to meet the mental health needs of this population, but very little research has explored telehealth for this population. Therefore, the current study on the preliminary efficacy of AFFIRM Online found that participants reported significantly reduced depressive symptoms and improved stress appraisal and coping skills compared to the waitlist control (Craig et al., 2021b), which was consistent with the study on the efficacy of AFFIRM (Craig et al., 2021a). In the survey of acceptability, 95.2% of participants indicated that they learned a lot from AFFIRM online and felt connected to the community, which was useful for dealing with stress. Therefore, besides providing clinical care, future research could also facilitate peer interaction during interventions to enhance the sense of community support and alleviate symptoms. In addition to cognitive and behavioral coping skill practice, the imi application was designed to promote identity affirmation by covering four guidance areas including gender, queerness, stress, and stigma (Bauermeister et al., 2022). At the four-week follow-up, participants in the treatment arm experienced the benefit of greater stress appraisal and reported significant reductions in depression and anxiety. Meanwhile, compared to the resource-only control arm, the treatment arm was more likely to express a positive experience and acceptability of the intervention and recommend it to their friends. A possible explanation for this might be that this application that had interactive capacities could be directly responsive to participants’ needs but participants in the control arm could only get access to the resource webpages.

Moreover, when many internet-based programs were proven to be significantly useful in decreasing depression and other mental issues for youth (Clarke et al., 2015), various studies have also been carried out to explore the effect of online programs with tutorials on sexual minority youth’s mental health. QueerViBE with trans youth provided interactive video tutorials which were a combination of theory relating to gender identity, queer theory, and masculinities together, and Martin reported that this intervention significantly lowered psychological distress and fostered more positive feelings about trans identity, having utility in decreasing depression symptoms (Martin, 2019). However, as this study’s sample is predominantly white participants, further research involving a larger and more racially diverse sample is necessary to evaluate the effectiveness of this intervention for transgender youth of color. Furthermore, some researchers have indicated that it is more difficult for sexual minorities living in rural areas to get access to mental health services (Barefoot et al., 2015), but online mindfulness-based stress reduction (Jabson Tree and Patterson, 2019), known as OMBSR, was delivered to LGB people in Appalachia, a conservative region in the United States. This research outcome, focusing on difference by gender, has reported that women’s perceived stress was 23% less than baseline and men’s perceived stress decreased by 40% at post-program. Besides, Brave Trails’ Virtual Camp has shown significant positive effects by not only promoting participants’ acceptance of affirming practices but also fostering connections within the group. It has led to an increase in self-esteem following the formation of friendships, subsequently resulting in a decrease in depressive symptoms. Accordingly, these findings suggest that S&F intervention could be a widely accessible and effective approach to assist LGBTQ+ youth in reducing the symptoms of depression and anxiety, thereby promoting positive mental health outcomes.

### 4.2. Implication for unstructured and formal intervention (U&F)

Despite an increase in web-based resources for LGBTQ+ youth (McInroy et al., 2019), there are few online adult-facilitated programs fostering resilience. Two studies have tried to explore the association between mobile and web apps which are supported by professionals and the outcome of mental health among young gender minorities. Q Chat Space allowed youth to join professional-facilitated support groups and get connected to people who share the same experience (Fish et al., 2022). The study findings suggested that it was an acceptable and feasible program for LGBTQ+ youth, and they were willing to discuss and share topics related to their lives and health, reporting better mental health than those who do not engage in the online community. Also, this study has shown that there is an urgent need to build a safe and supportive space or environment for these young people who have a strong desire to connect with LGBTQ peers.

Also, there had been no previous studies examining the use of a mobile intervention for depressive symptoms designed for young sexual minorities before the emergence of TODAY! app. The TODAY! app provided constantly refreshed real-time resources in response to users reporting a negative mood and offered daily coaching for coping skills. The qualitative analysis reported that all participants gave positive responses and expressed that this app effectively helped them relieve negative moods and decrease depressive symptoms. This finding that higher engagement with this intervention was linked to greater improvements in outcome variables is consistent with Baltierra’s examination of the role of engagement in determining a digital intervention’s efficacy (Baltierra et al., 2016). According to data analysis, further research should pay attention to presenting didactic content in multimedia formats, which could optimize learning (Abdulrahaman et al., 2020), instead of just text content. It should also provide more personalized and in-depth feedback on users’ mental health difficulties. Meanwhile, this study also suggested that the digital intervention would be expected to explore incorporating social networking features to help eliminate users’ sense of social isolation, which could be a crucial contributor to poor mental health outcomes (Garcia et al., 2020). Unlike S&F where LGBTQ+ youth can receive support from professional caregivers only during specific times, highly feasible U&F intervention could enable these young people to spontaneously access health education and treatment at any time when they may be experiencing negative moods or going through hardships, by using their mobile devices.

### 4.3. Implication for structured and informal intervention (S&I)

S&I intervention for sexual and gender minority youth has been represented by “serious games” or programs that utilize gaming features for such purposes as health improvements. SPARX is a form of computerized cognitive behavioral therapy in the serious game format in New Zealand, and previous studies have concluded that SPARX could be a promising alternative and treatment to usual care for adolescents with symptoms of depression (Fleming et al., 2012; Merry et al., 2012). Contrary to expectations, one study found that this self-help intervention was not as helpful for transgender youth compared to general users – transgender youth showed no significant change in depression scores after using the intervention (Lucassen et al., 2021), while general users reported significant improvements in their PHQ-A (Patient Health Questionnaire-modified for Adolescents) scores. A possible explanation for disappointingly poor completion rates and no change in the mental health outcome among transgender users might be a lack of representation of gender-diverse characters and no tailored or specific e-therapies to treat depression in transgender adolescents in this program. Therefore, SPARX was developed by researchers and clinicians into a specially adapted version of Rainbow SPARX for sexual and gender minority youth (Lucassen et al., 2015). In this study, sexual and gender minority participants reported decreased depressive and anxiety symptoms pre- to post-intervention, and more than 80% completing all the modules indicated that the game would be accepted by other peers. Nevertheless, this finding contrasts with a later qualitative study that reported relatively low rates of acceptability for Rainbow SPARX among LGBT+ youth in the United Kingdom (Lucassen et al., 2018). Participants indicated that the game had inadequate LGBTQ+-specific and up-to-date resources. They also pointed out issues with the graphics, speed, and controls of the game program, which were not very attractive to young people. On the other hand, because this digital game intervention was created in New Zealand, cultural mismatch contributed to dissatisfaction about the game from UK users and then led to less desirable mental health outcomes. It can thus be suggested that further research designing similar game interventions should consider a broader range of geographical areas and cultural contexts during development. Furthermore, many studies have underscored the importance of connectedness for psychological improvements in sexual and gender minority youth (Mendlein, 2016; Garcia et al., 2020; Montagno and Garrett-Walker, 2022). However, without interpersonal interaction and limited ability to detect users’ clinical state, this game intervention may be better delivered as a supplement, rather than an alternative, to therapist-guided treatment for LGBTQ+ youth (Strauss et al., 2019).

In the future, it is critical for researchers to leverage these technological advances in a culturally sensitive, clinically effective, and user-oriented manner. This could potentially involve the use of adaptive algorithms that adjust the intervention to the user’s progress and responses, thereby providing a more personalized and effective therapeutic experience. Also, the development of such interventions should involve extensive usability testing to ensure the systems are user-friendly, engaging, and suitable for the target population. As we move forward, it will be crucial to incorporate the perspectives of the LGBTQ+ community throughout the development process to ensure interventions are inclusive, representative, and most importantly, effective.

### 4.4. Implication for unstructured and informal intervention (U&I)

As an unstructured and informal intervention, social media use has become almost ubiquitous among LGBTQ+ youth in daily life. Young people use social networking sites to create and maintain social relationships, obtain various information, and for entertainment and relaxation (Whiting and Williams, 2013), Meyer suggested social support from communities could help sexual minority to buffer against minority stress and mental disorders (Meyer, 2003). For example, Craig and her colleagues have concluded that online media could empower LGBTQ+ youth to feel stronger through positive storylines, active communities, and support from celebrity role models and provide them with mental and emotional relief (Craig et al., 2015). Indeed, longitudinal research has reported that in LGB+ participants, online experiences of acceptance were negatively associated with depression and anxiety, protecting them from poor mental health outcomes (Pellicane et al., 2021).

However, social media as a “moderation-free” space also poses a threat to LGBTQ+ youth’s mental health because this population may be experiencing more discrimination and cyberbullying (Lucassen et al., 2018; Berger et al., 2021). A quantitative study examining Facebook use of LGBTQ+ young adults has suggested that cyberbullying and victimization showed a sizeable effect on psychological distress for LGBTQ youth (McConnell et al., 2017). Further, Vogel, Ramo, Prochaska, Meacham, Layton, and Humfleet have indicated that social media use for gender minority youth could become problematic when it interferes with functioning, and participants with greater problematic social media use had greater depressive symptoms and internalized stigma (Vogel et al., 2021).

Hence, to ensure that social media becomes an effective and positive intervention for helping young people improve their well-being, computer science is crucial in harnessing the potential of social media as a digital intervention tool for improving mental health outcomes among LGBTQ+ youth, while also mitigating its risks. Firstly, implementing machine learning algorithms for content moderation could be instrumental in identifying and filtering out harmful content, thereby reducing the occurrence of online harassment and hate speech. Secondly, artificial intelligence can also play a key role in providing personalized and adaptive online mental health support. This could include tailored recommendations for community resources, moderated discussion groups, and other mental health services that are most relevant to the individual’s identity and experiences. Moreover, the development of digital literacy programs, including education on online identity management and digital security, should be prioritized. For instance, young people could be taught how to manage privacy settings, identify and report inappropriate or harmful content, and develop resilience strategies when facing negative online experiences. Lastly, fostering collaborations between researchers, clinicians, computer scientists, and social media platforms can be key in developing technologies and algorithms that not only protect LGBTQ+ youth but also foster a more inclusive and supportive online environment. For instance, the use of natural language processing can help in detecting subtle forms of online harassment or discrimination, and machine learning algorithms could be trained to better understand the specific needs and vulnerabilities of the LGBTQ+ community. With the appropriate safeguards and supportive resources, social media can become an empowering platform for LGBTQ+ youth, fostering resilience, promoting well-being, and providing a sense of community and belonging.

### 4.5. Future direction

A discussion of four intervention streams showed that S&F and U&F have made significant progress in improving the mental health of LGBTQ+ youth, resulting in reduced depression and stress levels and increased coping skills. Although S&I and U&I interventions have shown potential effectiveness, they still present some uncertainties. Given their features, S&I and U&I could serve as valuable supplements to healthcare, strengthening the impact of therapist-guided treatments. Both the majority of included studies and previous research have emphasized the importance of social connections and community engagement for beneficial health outcomes (McDonald, 2018; Fish, 2020; Garcia et al., 2020). Consequently, platforms such as serious games and social networking sites could provide safe spaces for youth to connect with the community, access health resources, and share their experiences under their chosen “real identities.” To maximize the mental health benefits of these interventions, professional guidance and online content moderation are needed (Dias Oliva, 2020; Dias Oliva et al., 2021), thereby reducing the prevalence of problematic internet use.

Notably, none of the studies on digital intervention leveraged advanced technology such as virtual reality (VR) or the “Metaverse” to deliver healthcare to LGBTQ+ youth. VR, a technology that creates a computer-generated world for users to interact with, through real-time computer images, sounds, and other sensory inputs, offers a sense of immersion in the virtual environment. A systematic review of evidence between 2012 and 2015 featuring 24 RCT studies suggested that VR was effective for individuals with severe mental health outcomes (Valmaggia et al., 2016). A prior study also concluded that virtual environments are adaptable, programmable, and compatible with established psychological theories and practices (Gregg and Tarrier, 2007). VR’s synchronous models can fulfill the requirements for interaction and personalization, which are essential in digital mental interventions for LGBTQ+ youth, ensuring a consistent experience (Bell et al., 2020). For instance, therapists can interact with patients in real-time as they would in in-person consultations (Tan et al., 2022), monitor their clinical condition and emotional responses, and tailor treatment programs.

In addition, the Metaverse’s potential for facilitating client-professional interactions through customized 3D avatars may offer an enhanced experience compared to existing VR platforms (Usmani et al., 2022). Immersive spaces can also accommodate group and collaborative therapy sessions (Thomason, 2021), a mainstay in the current digital mental health approaches while ensuring privacy. Moreover, given the need for social connections and community support among LGBTQ+ youth, as highlighted in this review, the Metaverse could simulate real-life interactions through “avatars” (Maloney, 2021; Oh et al., 2022), providing socialization opportunities without the risk of depression from isolation. Even serious games, a type of digital health intervention, could be upgraded within the Metaverse, and a 3D immersive setup comparable to real-life experiences could be attractive to youth (Maloney, 2021). However, due to the cost and other constraints of the required devices, digital interventions delivered via VR and the Metaverse are not yet widespread. Still, scholars should continue exploring the effectiveness of VR and Metaverse-based interventions in addressing mental health disparities among LGBTQ+ youth.

## Data availability statement

The original contributions presented in the study are included in the article/supplementary material, further inquiries can be directed to the corresponding author.

## Author contributions

YL, XW, and WG: conceptualization, methodology, data collection, and draft write-up. HF and YW: project administration, data analysis, and interpretation. YL, HF, XW, and YW: proofreading or editing. All authors contributed to the article and approved the submitted version.

## Conflict of interest

XW was employed by Sage IT Consulting Group.

The remaining authors declare that the research was conducted in the absence of any commercial or financial relationships that could be construed as a potential conflict of interest.

## Publisher’s note

All claims expressed in this article are solely those of the authors and do not necessarily represent those of their affiliated organizations, or those of the publisher, the editors and the reviewers. Any product that may be evaluated in this article, or claim that may be made by its manufacturer, is not guaranteed or endorsed by the publisher.
